# Enzymatic Reactions in Near Critical CO_2_: The Effect of Pressure on Phenol Removal by Tyrosinase

**DOI:** 10.3390/ijms10125217

**Published:** 2009-12-01

**Authors:** Priscilla Amaral, Daniela Garcia, Miguel Cardoso, Marisa Mendes, Maria Alice Coelho, Fernando Pessoa

**Affiliations:** 1 School of Chemistry, Universidade Federal do Rio de Janeiro, CT, Bl. E, Cidade Universitária, 21949-900, Rio de Janeiro, RJ, Brazil; E-Mails: pamaral@ee.ufrj.br (P.A.); danigarcia13@yahoo.com.br (D.G.); alice@eq.ufrj.br (M.C.); 2 Chemical and Biological Engineering Department, Instituto Superior Técnico, Avenida Rovisco Pais, 1049-001 Lisboa, Portugal; E-Mail: miguel.cardoso@ist.utl.pt (M.C.); 3 Chemical Engineering Department, Universidade Federal Rural do Rio de Janeiro, BR 465, Km 7, 23890, Seropédica, RJ, Brazil; E-Mail: marisamf@ufrrj.br (M.M.)

**Keywords:** tyrosinase, supercritical CO_2_, enzymatic reaction, phenol

## Abstract

The use of enzymes in supercritical CO_2_ (SCCO_2_) has received extensive attention in recent years. Biocatalysts have the advantage of substrate specificity and SCCO_2_ offers several advantages over liquid solvents. This work deals with the utilization of SCCO_2_ as a medium for the enzymatic removal of phenol from aqueous solutions using tyrosinase. Since the presence of oxygen is crucial for the enzyme-catalyzed oxidation, the substantial solvating power of SCCO_2_ makes it a promising medium for such reactions. The conversion of phenol was higher at 10 MPa. Under near critical conditions (7 MPa, 35 °C), the addition of air at 5 × 10^5^ Pa of pressure improved phenol removal.

## 1. Introduction

**Scheme 1.**
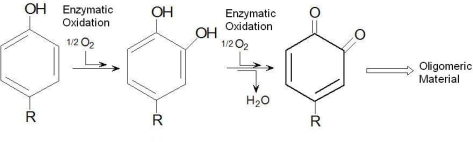
Phenol oxidation by polyphenol oxidase.

Phenols are toxic pollutants found in industrial wastewaters and that pose several risks to human health. Tyrosinase (EC 1.14.18.1) is a polyphenol oxidase found in several life forms, including the mushroom *Agaricus bisporus*, with great potential as a biocatalyst for applications involving biomodification of phenols or bioremediation of phenol-polluted water [1]. Kameda *et al*. [2] achieved 90% phenol removal in a synthetic phenol solution using mushroom tyrosinase. This enzyme requires molecular oxygen as a substrate, as described in Scheme 1, which shows the enzymatic conversion of monophenols that involves their hydroxylation to form *o*-hydroquinones, and then, dehydrogenation of the quinones to form *o*-benzoquinones. The benzoquinones finally undergo a non-enzymatic polymerization to yield water-insoluble substances.

The low oxygen solubility in aqueous media could be a limiting factor for the enzyme-catalyzed oxidation by tyrosinase as the presence of oxygen is required, but increasing the oxygen content of the system by increasing the rate of aeration may shear inactivate the enzyme. Also elevated oxygen partial pressures may be toxic and inactivate the enzyme.

Supercritical fluids (SCFs) have been used in biocatalysis to great advantage. The first reports on the use of SCFs in biochemical reactions were published in 1985 [3,4]. Hammond *et al*. [3] have proved that tyrosinase could be used in SCCO_2_. These preliminary studies indicated the possibility of enzymatic reactions in SCFs as the enzymes studied were able to retain their activity and stability in non-aqueous media. Since then, the use of supercritical CO_2_ (SCCO_2_) as a solvent for enzyme-catalyzed reactions has been a matter of considerable research interest because of its high diffusivity, low viscosity and low surface tension that can accelerate mass-transfer-limited enzymatic reactions [5]. Moreover, the use of SCFs does not have the drawback of leaving solvent residues in the reaction products because SCF solvents are gases under atmospheric conditions. In particular, the low toxicity and reactivity of SCCO_2_ make it attractive as non-aqueous reaction medium [6], as well as the low supercritical conditions (critical temperature, Tc = 31.04 °C and critical pressure, Pc = 7.38 MPa). The most exploited property of SCCO_2_ in the present work is its miscibility with other gases, such as oxygen, in the enzyme-catalyzed oxidation of phenol.

## 2. Results and Discussion

All reactions were performed in a two-phase (gas/liquid) reaction system with 210 mL of reaction medium in a 300-mL reactor. Preliminary studies were conducted in the apparatus shown in the Experimental Section at near critical conditions (35 °C, 7.00 MPa) to observe the performance of tyrosinase in the presence of CO_2_. After three hours, the enzymatic reaction under these conditions led to a 30% phenol removal. The presence of a limiting concentration of oxygen might be the reason for this low removal as the SCOO_2_ reactor is closed, avoiding the entrance of oxygen.

**Figure 1.**
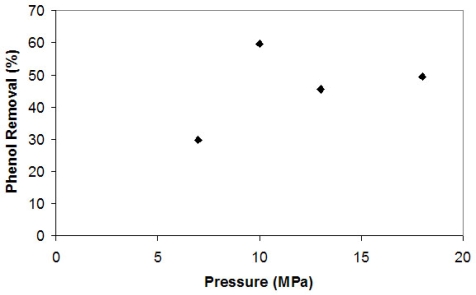
Phenol removal by tyrosinase in different CO_2_ pressures.

By raising the CO_2_ partial pressure at 35 °C, it achieves supercritical conditions above 7.38 MPa, leading to a volumetric expansion in the liquid, which allows a higher solubility of other gases, such as the oxygen present in the reactor. Figure 1 shows the results of phenol removal by tyrosinase under supercritical conditions at different pressures after a three hour reaction.

Initially, there was a raise in phenol removal as the partial pressure increased and then a slight reduction with increasing CO_2_ partial pressure. There seems to be an optimum condition related to the supercritical CO_2_. Probably, the results obtained were due to the solubility of both substrates and products in the SCCO_2_. The solubility of water in high pressure CO_2_ raises drastically at values near 10.0 MPa [6]. Therefore, phenol removal increases in this pressure range as the amount of oxygen raises, assuming the solubility in water is constant.

**Figure 2.**
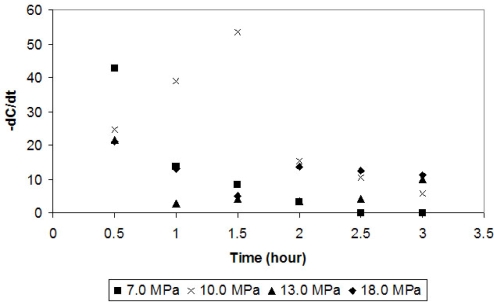
Phenol instantaneous consumption rate by tyrosinase in different pressures.

In order to study the progress of the enzymatic reaction, phenol instantaneous consumption rate (-dC/dt) was calculated. In Figure 2 it is possible to observe that for most pressure conditions the instantaneous phenol consumption rate decreases with time. This might happen because of enzyme inhibition by the product or the consumption of the oxygen in the reactor. A major drawback in the application of tyrosinase is the reaction product inhibition (suicide inactivation) exhibited by this enzyme [7]. Atlow *et al.* [1] used soluble tyrosinase to remove phenol from an aqueous synthetic waste solution; up to 99% conversion of the phenol was obtained, although, at higher concentrations (1.0 g/L), conversion was limited by inactivation of tyrosinase, likely by quinones formed during the reaction. Sun *et al*. [8] reported significant inactivation of soluble tyrosinase when phenol levels exceeded 0.05 g/L, likely because the quinones reacted with the free amino groups of the enzyme.

Under the best condition for phenol removal (10.0 MPa), there is a different behavior. Phenol instantaneous consumption rate raises until 1.5 hours of reaction (Figure 2). The solubility of the product (in supercritical phase) that inactivates the enzyme must be different from the solubility of the enzyme (in supercritical phase) under this condition and, therefore, there is a greater yield of the reaction under this condition. Afterwards, there is a reduction of the phenol consumption rate, which might be due to the exhaustion of oxygen.

**Figure 3.**
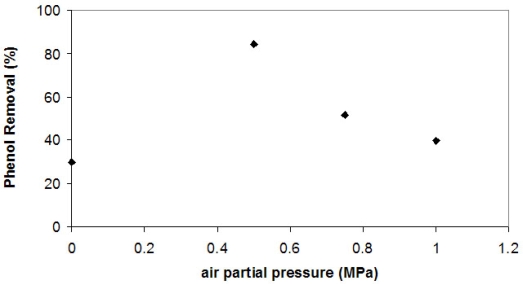
Phenol removal by tyrosinase CO_2_ at near critical conditions (35 °C, CO_2_ partial pressure = 7.0 MPa) at different air partial pressure.

In order to raise the oxygen content of the reactor, an air compressor was added to the experimental apparatus shown in the Experimental. The air was added to the reactor after CO_2_ reached a partial pressure of 7.00 MPa. Three different air partial pressures were used: 0.50, 0.75 and 1.00 MPa. The results are presented in Figure 3.

The addition of air improved phenol removal by tyrosinase (from no air to 0.50 MPa), showing that one of the limitations of the reaction was the absence of oxygen. However, there is a maximum phenol removal with an air partial pressure of 0.50 MPa, and higher pressures reduced phenol removal. Table 1 shows phenol instantaneous consumption rate (-dC/dt) of these experiments at the first 30 minutes of reaction. In comparison to the experiments without addition of air (Figure 2), these rates are higher. Raising air partial pressure further (1.00 MPa), the initial phenol removal rate as well as the phenol removal diminish. Presumably, with more air available suicide inactivation becomes the limiting factor of the reaction as the phenol removal rate is slower.

## 3. Experimental Section

Tyrosinase crude extract was prepared from common mushrooms (*Agaricus bisporus*) purchased in a local market (Rio de Janeiro, RJ, Brazil). The extraction consisted in triturating the mushrooms with different volumes of pre-cooled acetone, filtering the pulp, freezing it for 24 hours, suspending in 300 mL of distilled water, incubating the suspension overnight in a freezer, centrifuging the resultant suspension, and recovering the first extract. The suspension in water and centrifugation steps were repeated twice to obtain two more extracts [9].

Tyrosinase activity was measured with a HACH DR/4000 UV-Vis spectrophotometer: a sample of the enzyme was added to a l-tyrosine solution (1.2 mM) in a phosphate buffer (0.2 M, pH 6.0). A linear increase in absorbance at 280 nm was noted [10]. Phenol was determined by a colorimetric assay [11] based on the absorbance at 500 nm caused by the reaction between phenol, 4-aminoantipyrine and potassium ferricyanide. Phenol Removal was calculated by the equation below:

(1) $$\text{Phenol}\;\text{Removal}=\frac{\left(\text{Cf}-\text{Ci}\right)}{\text{Ci}}*100\%$$

where C stands for phenol concentration, and f and i indicate final and initial, respectively.

**Figure 4.**
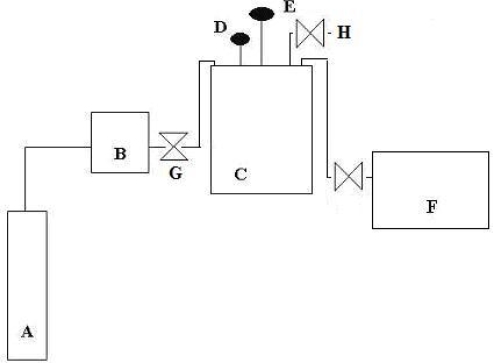
Schematic illustration of the experimental apparatus: A, CO_2_ cylinder; B, syringe pump; C, reactor; D, temperature controller; E, pressure transducer; F, air compressor, G cold trap; H, sampler tube and valve.

The experimental set-up, shown in Figure 4, basically consisted of a CO_2_ cylinder, a high pressure pump (ISCO 260D) and a 300-mL reactor (Autoclave Engineers, model 300BG) equipped with a mechanical stirrer (Mag-neDrive II), a heating mantle, an internal cooling loop capable of maintaining the temperature constant within 0.1°C and a pressure transducer. The enzymatic reaction involved a 210 mL-aqueous reaction mixture with 200 U/mL of tyrosinase and 100 mg/L of phenol that were placed in the reactor. The free-volume inside the reactor (90 mL) containing air, was the source of oxygen for the reaction. Afterwards, the reactor was closed, flushed and pressurized with CO_2_. The substrates and enzyme were then continuously mixed with an agitation level of 1,250 rpm in order to provide a proper homogenization of the reacting mixture. The reaction time was 3 h and samples were withdrawn every 30 minutes.

## 4. Conclusions

Under supercritical conditions, 10.0 MPa was the best pressure for phenol removal, but oxygen was still the limiting factor. With the addition of air (0.5 MPa), it was possible to achieve 84.4% of phenol removal in near critical CO_2_ conditions in a closed reactor, showing the potential to use this process as a regenerator of aqueous effluents when phenol is present. One great advantage of this result is that the enzyme recovery becomes easier using SCCO_2_ as reaction medium than in conventional processes, since each medium constituent has a different solubility in SCCO_2_. By varying the pressure of the system it should be possible to recover each constituent separately.

## Figures and Tables

**Table 1.** Phenol instantaneous consumption rate (-dC/dt) in CO_2_ near critical conditions (35 °C, CO_2_ partial pressure = 7.0 MPa) for different air partial pressure at the first 30 minutes of reaction.

**Air Partial Pressure (MPa)**	**-dC/dt (ppm/h)**
0.10	42.8
0.50	194.6
0.75	208.5
1.00	37.5
